# A showcase of future plant biology: moving towards next-generation plant genetics assisted by genome sequencing and systems biology

**DOI:** 10.1186/gb4176

**Published:** 2014-05-23

**Authors:** Insuk Lee

**Affiliations:** 1 Department of Biotechnology, Yonsei University, Seoul 120-749, Korea

## Abstract

A report on the Cold Spring Harbor Asia conference on Genome Assisted Biology of Crops and Model Plant Systems Meeting, held in Suzhou, China, April 21–25, 2014.

## Meeting report

Plant genomes are generally much larger and more complex than those of animals, encoding molecular circuits of high sophistication and robustness to control physiology. Due to the immobility of plants, their large and complex genomes have evolved to encode an organism adapted to a constantly changing environment. In addition to the natural selection process, the genomes of domesticated crops have been selected for desired traits by breeding. Thus far, the adaptability of plants and selective breeding have created crops to match the ever-increasing human demand for food and energy. However, given the explosive growth of the global population and the concomitant decrease in the amount of arable land, current breeding strategies alone will not be able to maintain a sufficient supply of plant-derived food and energy sources for the entire world. Therefore, understanding and engineering molecular networks that control plant phenotypes, as well as the identification of new genomic markers for selection of these phenotypes, will be crucial to begin the next green revolution.

Embracing these impending agricultural challenges, over 100 participants gathered at the Cold Spring Harbor Asia conference Genome Assisted Biology of Crops and Model Plant Systems Meeting to discuss recent progress in a diverse array of plant science topics. Four major themes emerged during the meeting, including the extended applications of next-generation sequencing technologies to explore genetic elements and their networks to control plant phenotypes, ways to integrate multiple types of -omics data and genetics data, moving from model plants to crops, and challenges to the quantitative modeling of complex traits.

## Next-generation genetics powered by resequencing

The conference discussions covered many crop species, including rice, maize, foxtail millet, wheat, sorghum, oil palm, tomato and cucumber. Decoding their reference genomes has resulted in a new era of genome-assisted crop genetics. Recently, resequencing of a large number of accessions, followed by population-genomics analysis, has revealed genetic loci for the domestication of and improvements in crops such as rice and maize. Sanwen Huang (Chinese Academy of Agricultural Science, China) demonstrated the feasibility of resequencing-assisted genetics in cucumber, which is also a model for plant vascular biology and sex determination. Deep resequencing of 115 cucumber lines, sampled from 3,342 accessions worldwide, identified 112 domestication sweeps. Interestingly, one of these regions contains a gene for the loss of bitterness, an essential domestication trait of cucumber. Single-nucleotide polymorphism (SNP) markers identified from resequencing are generally tested for their association with traits of agricultural importance, such as plant growth and development, and metabolites are often crucial for such traits. Therefore, genome-wide association of SNPs with metabolic traits should provide new insights about the metabolic pathways essential for plant growth and development. Jie Luo (Huazhong Agricultural University, China) presented the results of a metabolome-based genome-wide association study (mGWAS) of maize and rice using a new, widely targeted metabolomics strategy that simultaneously detects hundreds of primary and secondary metabolites. The mGWAS successfully identified candidate genes involved in crucial metabolic traits.

Robert Martienssen (Cold Spring Harbor Laboratory, USA) introduced a variety of resequencing-assisted genetics strategies to discover key genes for oil palm domestication and breeding. Oil from fruit and seeds provides approximately 45 % of edible oil globally, and palm oil is the most efficient source of biodiesel. Shell thickness controls oil yield, and oil palms with thin-shelled fruit have been selected by breeders. To clone the genes responsible for this trait, Martienssen’s team first mapped the genomic interval for shell thickness from hundreds of inbred lines derived from self-pollination of a thin-shelled accession. Genomic regions with high homozygosity may contain genes selected during domestication and breeding. Given this hypothesis, genomic regions were scored for homozygosity using SNPs from resequencing data. Using the ‘homozygosity mapping by resequencing’ approach, his team successfully cloned the *SHELL* gene.

Resequencing-assisted forward genetics has proven useful in model organisms, including *Arabidopsis*. In this approach, a mutant population is generated by treatment with ethyl methanesulfonate (EMS), and then mutations that disrupt genes for the trait of interest are identified by resequencing the mutant genomes. Brian Dilkes (Purdue University, USA) demonstrated that a similar approach would also be effective in crops by cloning genes affecting the accumulation of insect defense compounds in sorghum. He also reported the discovery of error-prone regions of the sorghum genome, perhaps due to genome assembly quality. This finding demonstrated the importance of systematic cataloging of variants from a large number of resequenced genomes to improve future applications of resequencing-assisted genetics.

## Unraveling molecular networks that control plant traits

Most plant phenotypes are regulated by highly complex and robust molecular networks. Unraveling the network structure and identifying its key components will provide effective targets for crop engineering. During the conference, extended models for many such networks derived from systematic and integrative approaches were presented. *Lotus japonicus* is a model plant that is used to study regulatory networks controlling nitrogen fixation by symbiotic nodulation. Masayoshi Kawaguchi (National Institute for Basic Biology, Japan) reported an extended model of a long-distance root-to-shoot-to-root signaling network that controls nodulation. Multi-omics approaches elucidated post-translational modifications for a key signaling molecule that functions in long-distance interactions with other signaling network molecules. Another relatively small but sophisticated model network presented at the conference was for age-dependent cell death signaling in *Arabidopsis*. Hong Gil Nam (DGIST, Korea) presented data on a core senescence regulatory network mediated by *ORE1*, in which a highly robust regulatory circuit includes a trifurcate, feed-forward pathway, involving *ORE1*, *EIN2* and *miR164*. He also introduced his ongoing efforts to identify the functional and regulatory networks for temporal and spatial coordination of the age-dependent developmental transitions by integrating various multi-dimensional transcriptomes of the *Arabidopsi*s leaf during the entire plant life span.

Crop yields could be improved by engineering molecular networks that control the development of plant organs. Dirk Inze (Gent University, Belgium) discussed a key molecular network in determining the timing of the transition from cell division to cell expansion, which is relevant to final leaf size. Chromatin-remodeling complexes comprise the core network, and their dynamic interactions with transcriptional co-activators were detected by proteomics approaches. He also showed that overexpression of key components of the network can increase leaf size in *Arabidopsis*. Andrea Eveland (Cold Spring Harbor Laboratory, USA) presented a regulatory network that controls maize inflorescence architecture, reconstructed from the integrative analysis of spatiotemporal gene expression profiles and genome-wide binding loci of key regulators. The elucidated regulatory modules could go on to provide a molecular target for making improvements to organ architecture in cereal crops.

Larger-scale network modeling efforts were also discussed during the meeting. Thomas Brutnell (Donald Danforth Plant Science Center, USA) presented his recent efforts in the reconstruction of molecular networks for photosynthesis in rice, maize and a new model plant, *Setaria viridis* (green millet). Using integrative system-level approaches, including high-resolution leaf transcriptome profiling, his team is characterizing systems-level differences between a less-efficient C3 photosynthesis network and a more-efficient C4 photosynthesis network. Information from the study could be used to improve the photosynthetic systems of C3 crops in the future.

In contrast to the well-characterized molecular networks of each biological process, a genome-wide predictive network could provide a platform in which to study many pathways and interpathway organization. Such networks could also be used for the functional study of individual genes based on guilt-by-association principles or other network algorithms. Insuk Lee (Yonsei University, Korea) presented genome-wide functional gene networks for *Arabidopsis* and rice and described their use for the discovery of novel gene functions. He also proposed functional gene networks as efficient technical platforms for big-data plant biology to enable the analysis and integration of heterogeneous data from diverse public databases.

## New challenges in crop science

Studies of determining factors in transcriptional programs in plants have been conducted mainly in laboratories or greenhouses, rather than in the field. However, most crops are typically grown in complex, fluctuating environments. Takeshi Izawa (National Institute of Agrobiological Science, Japan) has developed a statistical model for the rice transcriptome in the field, revealing that gene expression dynamics can be predicted for changing temperatures. Such models accounting for environmental factors will help to translate genotype-to-phenotype associations discovered in the laboratory to agricultural traits of crops in the field, reducing gaps between laboratory models and field phenotypes.

Another new challenge in crop science is gaining improvements in the flavor quality of fruits. Harry Klee (University of Florida, USA) argued that traditional breeding programs have been biased towards yield, resulting in reduced flavors. He showed how to identify the molecules responsible for flavor quality using consumer-assisted selection programs. He demonstrated that not only taste, but also olfaction, are important for fruit flavor, and hundreds of chemicals can be used to improve flavor. Although the genetics of flavor appears complex, information from this study will help to promote the commercial value of fruits, as well as enhance a healthier diet in the future.

## Conclusions

Despite a long history of plant genetics, genotype-to-phenotype association mapping is still largely limited in plants owing to their immense genomic space and confounding effects of environmental factors. Advanced sequencing technologies, along with systems-genetics approaches, however, are promising for unveiling such complex relationships between genetic variations and phenotype differences in plants. Next-generation sequencing with improved accuracy and read length has resolved many technical barriers in traditional genetics approaches. In addition, high-throughput molecular profiles and advanced modeling technologies have elucidated the genetic networks around key regulatory genes for a specific phenotype, accounting for the effects of molecular changes on emergent phenotypes. Most, if not all, of the meeting participants agreed that we are entering a golden era of crop science because of advanced genomics technologies.

## Abbreviations

EMS: Ethyl methanesulfonate; mGWAS: Metabolome-based genome-wide association study; SNP: Single-nucleotide polymorphism.

## Competing interests

The authors declare that they have no competing interests.

